# Lipid Control in Patients With Type 2 Diabetes Mellitus: A Continuous Quality Improvement Study

**DOI:** 10.7759/cureus.85278

**Published:** 2025-06-03

**Authors:** Andreia G Sousa, Daniela de Azevedo, Suzie Leandro, Marta S Ferreira, Tiago A Correia, Mariana Sá, Diana Capela

**Affiliations:** 1 Family Medicine, Family Health Unit Famílias, Entre Douro e Vouga Local Health Unit, Santa Maria da Feira, PRT; 2 Family Medicine, Family Health Unit Novo Este, Entre Douro e Vouga Local Health Unit, Santa Maria da Feira, PRT; 3 Family Medicine, Personalized Healthcare Unit Novo Caminho, Entre Douro e Vouga Local Health Unit, Oliveira de Azeméis, PRT; 4 Family Medicine, Family Health Unit Flor d'Areosa, Região de Aveiro Local Health Unit, Aveiro, PRT

**Keywords:** cardiovascular risk, dyslipidemia, ldl-cholesterol, lipid-lowering therapy, therapeutic inertia, type 2 diabetes mellitus

## Abstract

Introduction

Type 2 diabetes mellitus (T2DM) is highly prevalent in Portugal, ranking among the highest in Europe, according to Organisation for Economic Co-operation and Development (OECD) data. Cardiovascular risk (CVR) control, particularly dyslipidemia management, is a key contributor to long-term health outcomes. This study aims to improve lipid management in T2DM patients, with the primary objective of achieving a 25% lipid control rate.

Methodology

A continuous quality improvement study was conducted in a Portuguese family health unit, involving independent random samples of patients with T2DM at each assessment period. The assessments were performed at three distinct time periods: January-June 2022, November 2023-April 2024, and May-October 2024. Interventions included team-based training workshops, monthly electronic reminders, visual aids, anonymous performance feedback, and collaborative consultations with nurses to support guideline-based lipid management. Low-density lipoprotein cholesterol (LDL-C) targets were less than 100, 70, or 55 mg/dL for moderate, high, or very high CVR. Non-high-density lipoprotein cholesterol (non-HDL-C) was used to assess lipid control when triglyceride levels were above 200 mg/dL, targeting below 135, 100, and 85 mg/dL for moderate, high, or very high CVR, respectively. Therapeutic inertia was defined as the absence of treatment adjustment despite evidence of inadequate lipid control, excluding cases where non-intensification was due to documented treatment refusal or poor adherence.

Results

Lipid control was achieved in 55 (20.4%) of 270 patients at baseline, rising to 60 (21.7%) of 276 in the second assessment and 90 (32.5%) of 277 in the final period, representing a 12.1 percentage point improvement (p<0.001). Among patients with high CVR, control increased from 45 (23.9%) to 61 (38.6%) and from 10 (12.2%) to 29 (24.4%) in those with very high CVR. A significant reduction in median LDL-C levels was also observed (p<0.001). Therapeutic inertia affected 199 (93.9%) patients initially, decreasing to 104 (73.8%) in the third assessment (p<0.001). Despite progress, a relevant proportion of patients remained without lipid-lowering therapy, although they did not meet recommended targets: 54 (25.2%) at baseline and 29 (15.5%) in the final period. Prescription of combination therapy with ezetimibe remained infrequent. Additionally, documentation of non-adherence and treatment refusal improved throughout the study.

Conclusion

The intervention showed improvement in lipid control and reduction in therapeutic inertia. Despite this progress, lipid control remained suboptimal, requiring ongoing education of healthcare professionals and patients to enhance CVR management and reduce cardiovascular events. Extending the intervention with further evaluations would help monitor long-term effectiveness. To enhance outcomes, future strategies should promote greater use of combination therapy, implement patient-specific approaches to improve adherence, and integrate clinical decision support tools to reduce therapeutic inertia. Biases such as inter-observer variability and the Hawthorne effect may have influenced the findings.

## Introduction

Cardiovascular diseases (CVD) remain the leading cause of morbidity and mortality on a global scale, including in Portugal, where, in 2022, approximately 26.5% of deaths were attributed to diseases of the circulatory system [1-3]. Dyslipidemia and type 2 diabetes mellitus (T2DM) are significant contributors to cardiovascular risk (CVR), especially through their combined effect in accelerating atherosclerosis, through mechanisms such as endothelial dysfunction and lipid accumulation [4-6]. T2DM, often accompanied by dyslipidemia, poses a major public health challenge, as most diabetes-related deaths stem from atherosclerotic disease, and the presence of dyslipidemia exacerbates insulin resistance in these patients [5,7], despite the availability of preventive measures [4]. This effect occurs through mechanisms such as lipotoxicity, oxidative stress, and low-grade inflammation caused by elevated free fatty acids and altered adipokine signaling [6]. While traditionally associated with older adults, both T2DM and dyslipidemia are increasingly becoming common in the pediatric and youth groups, contributing to the earlier onset of cardiovascular complications, including heart failure, and worse long-term outcomes [8]. These trends underscore the importance of effective lipid management, particularly low-density lipoprotein cholesterol (LDL-C) control, a crucial aspect of diabetes care, as it significantly reduces cardiovascular complications in patients with T2DM [5,6].

Nevertheless, the risk of CVD varies among individuals with T2DM. According to the 2021 European Society of Cardiology (ESC) Guidelines on CVD prevention, all individuals with T2DM are considered to be at least at moderate risk, with most classified as high or very high risk depending on factors such as diabetes duration and the presence of target organ damage and major risk factors [4]. Similarly, the 2023 American Diabetes Association (ADA) Standards of Care recommend incorporating risk-enhancing factors and established atherosclerotic cardiovascular disease (ASCVD) risk calculators to stratify patients and guide the intensity of lipid-lowering therapy. These assessments help determine whether a patient requires moderate- or high-intensity treatment, as recent guidelines set strict LDL-C targets based on individual CVR categories [9]. Recommended LDL-C targets are <100 mg/dL (<2.6 mmol/L) for individuals at moderate risk, <70 mg/dL (<1.8 mmol/L) for those at high risk, and <55 mg/dL (<1.4 mmol/L) for very-high-risk individuals, with a required reduction of at least 50% from baseline levels in patients at high and very high CVR. Following the assessment of CVR based on LDL-C levels, it is important to optimize non-high-density lipoprotein cholesterol (non-HDL-C) levels, particularly in patients with triglyceride concentrations >200 mg/dL. Recommended non-HDL-C targets are <130 mg/dL (<3.4 mmol/L), <100 mg/dL (<2.6 mmol/L), and <85 mg/dL (<2.2 mmol/L) for individuals at moderate, high, and very high CVR, respectively [4]. These guidelines directly informed the study design, particularly in the classification of CVR and the definition of lipid targets, ensuring alignment with current international standards.

Despite these most recent guidelines, achieving recommended lipid targets remains challenging. Data from the European SANTORINI study indicate that approximately 80% of high- and very-high-risk patients do not achieve LDL-C goals due to CVR underestimation and insufficient use of combination therapies [10]. Real-world evidence from international cohorts highlights a significant discrepancy between clinical guidelines and practice, with a notably low proportion of patients with T2DM achieving recommended LDL-C targets despite statin therapy [11,12]. In Portugal, observational studies indicate suboptimal lipid control among T2DM patients. One study conducted in several family health units (FHU) found that only 32.1% of patients achieved good lipid control after targeted interventions, which included educational sessions, audit and feedback, and development of decision support tools [13]. 

Collectively, these findings emphasize persistent gaps in lipid management among patients with T2DM worldwide and highlight the urgent need for the implementation of guideline-recommended therapeutic approaches. Interventions that stem from continuous quality improvement (CQI) studies, encompassing education, structured reminders, and interprofessional collaboration, are essential to enhance clinical outcomes. The primary objective of this study is to evaluate and improve lipid management in patients with T2DM within a Portuguese FHU, with the goal of achieving LDL-C targets in at least 25% of patients, according to their CVR category. This target was selected as a realistic yet ambitious threshold, based on its use as a quality benchmark in a previous Portuguese study [13] and considering that baseline control in our population was below this level.

## Materials and methods

This CQI study was designed as a repeated cross-sectional study conducted at the Family Health Unit (FHU) Famílias, part of the Entre Douro e Vouga Local Health Unit, located in Santa Maria da Feira, Aveiro, Portugal. It includes three assessment periods: an initial evaluation from January to June 2022, a second from November 2023 to April 2024, and a third from May to October 2024. The initial assessment served to establish a baseline of lipid control among patients with T2DM, with no intervention planned at that stage. Following internal discussion in late 2022, the team decided to implement a CQI project, leading to a preparatory phase that explains the gap between the first and second assessments. The second and third periods were subsequently conducted to evaluate the impact of the intervention.

The study population included adult patients diagnosed with T2DM, classified as T90 according to the International Classification of Primary Care, Second Edition (ICPC-2). A representative sample was calculated using the Raosoft® online calculator (Raosoft Inc., Seattle, Washington, United States), which applies the standard formula for estimating sample sizes for proportions in large populations. This tool determines the minimum required sample size for population surveys based on the desired confidence level, margin of error, and population size, according to the formula $n_{0}=\frac{Z^{2}\times p\times (1-p)}{e^{2}}$, where Z is the z-score corresponding to the desired confidence level (1.96 for 95%), p is the estimated population proportion, and e is the margin of error. As the true proportion of patients achieving lipid control was unknown at the outset, the default response distribution of 50% (p=0.5) was applied in the calculator. This value, which maximizes statistical variance, is widely used in health research when prior estimates are unavailable, as it provides a conservative and statistically robust sample size.

The formula above estimates the sample size for an effectively infinite population. However, when the target population is finite, as in this study, the Raosoft® calculator applies a correction factor to adjust the initial estimate. This adjustment is made using the formula $​​​​​​​n=\frac{n_{0}}{1+\frac{(n_{0}-1)}{N}}$, where n₀ is the sample size for an infinite population and N is the actual population size. This correction ensures that the calculated sample size accurately reflects the smaller, known population, resulting in a more precise and efficient sampling approach.

For each assessment period, a 95% confidence level, a 5% margin of error, and the actual population sizes (906, 966, and 989 patients, respectively) were entered into the calculator. The resulting minimum required sample sizes were 270, 276, and 277 participants, respectively, precisely matching the sample sizes obtained in each period. This alignment confirms that the samples were adequately representative of the target populations.

Following the sample size calculation, participants were selected through independent random sampling from the total eligible T2DM population in each assessment period. As different patients were included at each stage, there were no drop-outs or losses to follow-up. Inclusion and exclusion criteria were reapplied consistently to ensure comparability across samples. Exclusion criteria were the absence of a medical consultation during the study period, unavailability of a lipid profile result within the assessment timeframe, pregnancy, and triglyceride levels exceeding 400 mg/dL, as such cases reduce the accuracy of LDL-C estimation using the Friedewald formula and may compromise the validity of lipid target classification.

Collected data are summarized in Table 1, encompassing demographic information, clinical parameters, laboratory findings, disease duration, comorbidities, CVR classification, lipid control, pharmacological treatment for dyslipidemia, and therapeutic inertia.

**Table 1:** Description of study variables BMI: body mass index; BP: blood pressure; CVD: cardiovascular disease; CVR: cardiovascular risk; ESC: European Society of Cardiology; GFR: glomerular filtration rate; HbA1c: glycated hemoglobin; HDL-C: high-density lipoprotein cholesterol; ICPC-2: International Classification of Primary Care; LDL-C: low-density lipoprotein cholesterol; T2DM: type 2 diabetes mellitus; CKD-EPI: Chronic Kidney Disease Epidemiology Collaboration

Variable	Type	Description	Determination
Gender	Nominal	Female or male	Documented in the patient's demographic information
Age	Continuous	Reported in years	Documented in the patient's demographic information
BMI	Continuous	Reported in kg/m²	Calculated and documented in the clinical records within the T2DM management program
BMI classification	Nominal	Underweight, normal, overweight, or obese	Underweight <18.5 kg/m²; normal 18.5-24.9 kg/m²; overweight 25-29.9 kg/m²; obese ≥30 kg/m²
Smoking history	Nominal	Smoker or non-smoker	Documented in the clinical records within the T2DM management program
Alcohol consumption	Continuous	Reported in g per week	Documented in the clinical records within the T2DM management program. Includes conversion from standard drinks (10-12 g of pure alcohol per unit)
Harmful alcohol consumption	Nominal	Yes or no	Considered harmful if >100 g/week, based on 2021 ESC guidelines on CVD prevention
BP	Continuous	Reported in mmHg	Mean of the lowest two of three measurements, five minutes apart, in a seated position, using an automatic sphygmomanometer
HbA1c	Continuous	Reported in %	The most up-to-date laboratory test registration
Creatinine	Continuous	Reported in mg/dL	The most up-to-date laboratory test registration
GFR	Continuous	Reported in mL/min/1.73 m²	Calculated using the 2021 CKD-EPI creatinine equation (race-free), based on serum creatinine, age, and sex
Duration of T2DM	Continuous	Reported in years	Calculated by subtracting the year of diagnosis from the year of assessment, using the ICPC-2 code T90 (T2DM) documented in the patient's problem list
Retinopathy	Nominal	Yes or no	Presence of ICPC-2 code F83 (retinopathy) in the patient's problem list
Nephropathy	Nominal	Yes or no	GFR <60 mL/min/1.73 m² or presence of ICPC-2 code U88 (glomerulonephritis/nephrosis) in the patient's problem list or albumin/creatinine ratio >30 mg/g in two consecutive separate urine tests
Neuropathy	Nominal	Yes or no	Presence of ICPC-2 code N94 (neuritis/peripheral neuropathy) in the patient's problem list
Coronary heart disease	Nominal	Yes or no	Presence of at least one of the ICPC-2 codes K74 (ischemic heart disease with angina), K75 (acute myocardial infarction), or K76 (ischemic heart disease without angina) in the patient's problem list
Cerebral vascular disease	Nominal	Yes or no	Presence of at least one of the ICPC-2 codes K89 (transient cerebral ischemia), K90 (thrombosis/cerebrovascular accident), or K91 (cerebrovascular disease) in the patient's problem list
Peripheral arterial disease	Nominal	Yes or no	Presence of ICPC-2 code K92 (atherosclerosis/peripheral vascular disease) in the patient's problem list
LDL-C	Continuous	Reported in mg/dL	The most up-to-date laboratory test registration
Total cholesterol	Continuous	Reported in mg/dL	The most up-to-date laboratory test registration
HDL-C	Continuous	Reported in mg/dL	The most up-to-date laboratory test registration
Triglyceride	Continuous	Reported in mg/dL	The most up-to-date laboratory test registration
CVR	Nominal	Moderate, high, or very high	Based on 2021 ESC guidelines on CVD prevention, according to T2DM duration, target organ damage, GFR, BP, lipid values, comorbidities, and presence of familial hypercholesterolemia
Lipid control	Nominal	Within lipid target or out of lipid target	According to CVR: <100 mg/dL (moderate), <70 (high), and <55 (very high). In patients with triglyceride levels >200 mg/dL, non-HDL-C targets were used: <130 mg/dL, <100 mg/dL, and <85 mg/dL for individuals at moderate, high, and very high CVR, respectively
Dyslipidemia treatment	Nominal	Various treatment combinations, poor adherence, refusal, or intolerance	Documented in clinical records
Reason for treatment refusal or poor adherence	Nominal	Forgetfulness, fear of side effects, fear of dependence, low health literacy, lifestyle decisions, cost, or unknown	Categorized from clinical records based on documented reasons
Therapeutic inertia	Nominal	Yes or no	Documented in the clinical records and defined as the absence of treatment adjustment despite evidence of inadequate lipid control. Cases where lack of adjustment was due to patient refusal or non-adherence were excluded

Statin intensity classification followed the 2018 American Heart Association (AHA)/American College of Cardiology (ACC) cholesterol guideline, which categorizes statins based on expected LDL-C reduction [14], as presented in Table 2.

**Table 2:** Statin therapy intensity LDL-C: low-density lipoprotein cholesterol

	High intensity	Moderate intensity	Low intensity
LDL-C lowering	≥50%	30-49%	<30%
Statins	Atorvastatin 40-80 mg	Atorvastatin 10-20 mg	Simvastatin 10 mg
	Rosuvastatin 20-40 mg	Rosuvastatin 5-10 mg	Pravastatin 10-20 mg
		Simvastatin 20-40 mg	Lovastatin 20 mg
		Pravastatin 40-80 mg	Fluvastatin 20-40 mg
		Lovastatin 40-80 mg	
		Fluvastatin XL 80 mg	
		Fluvastatin 40 mg twice daily	
		Pitavastatin 1-4 mg	

Data were extracted from electronic medical records (SClínico®) and securely stored in encrypted databases. A descriptive analysis was conducted, using absolute (n) and relative frequencies (%) for categorical variables. Continuous variables were summarized using means and standard deviations or medians and interquartile ranges (IQR) when the distribution was non-normal. Normality was assessed using histogram visualization and the Shapiro-Wilk test.

Inferential statistical analyses were conducted to assess the effectiveness of the intervention over the three assessment periods. The Mann-Whitney U test was used to compare non-normally distributed continuous variables, such as LDL-C, between independent samples. The z-test for proportions was applied to evaluate differences in categorical outcomes, including the proportion of patients achieving lipid control and those affected by therapeutic inertia. These tests were chosen for their appropriateness in comparing independent groups and evaluating statistically significant differences between assessments. A two-tailed significance level of p<0.05 was adopted. All analyses were performed using IBM SPSS Statistics for Windows, Version 29.0.2.0 (Released 2023; IBM Corp., Armonk, New York, United States).

Quality indicators focused on lipid control among patients with T2DM, specifically the proportion of individuals achieving LDL-C or non-HDL-C (if applicable) targets based on their CVR category. The quality standard was defined as follows: an improvement to ≥35% was classified as "very good", 30-34.9% "good", 25-29.9% "sufficient", and <25% "insufficient". The quality indicator thresholds were defined by the study authors based on local clinical context and benchmarking expectations, informed by a previous Portuguese study [13] and the need for achievable, incremental improvement targets.

Interventions included two in-person workshops conducted between assessment periods for physicians and nurses, focusing on CVR stratification and lipid control strategies. Participation was voluntary but strongly encouraged. Monthly electronic reminders were sent to the clinical team, reinforcing LDL-C and non-HDL-C targets as well as therapeutic options. Visual memory aids were placed on clinical office computers, and pocket-sized guides summarizing CVR categories and pharmacological approaches were distributed. Interprofessional collaboration was promoted during joint consultations, wherein nurses systematically identified and alerted physicians about patients not meeting therapeutic targets. Patients with T2DM received printed, individualized LDL-C targets at each visit. Health education outreach included informational pamphlets and infographics on food label reading, which were distributed at community events and consultations. Throughout the study, anonymous performance feedback was provided to the team using symbolic representations for each physician. A brief, non-validated anonymous survey was distributed to the healthcare professionals at the unit, with a response rate of 71.4%. The results were analyzed descriptively and used to identify common doubts and barriers, which were then discussed during interprofessional meetings.

This study was approved by the Ethics Committee of the Northern Regional Health Administration (approval number: CE/2024/21). Patient data confidentiality was strictly maintained. All activities complied with good clinical practice guidelines. As only anonymized retrospective data were used, informed consent was waived.

## Results

Sample characteristics

After determining the required sample size to ensure representativeness of patients diagnosed with T2DM at FHU Famílias, the final study samples included 270 patients (out of 906) in the first assessment, 276 (out of 966) in the second, and 277 (out of 989) in the third. Exclusion criteria were applied prior to analysis, and only patients meeting all eligibility requirements were included. The demographic and clinical characteristics of the participants across the three assessments are summarized in Table 3.

**Table 3:** Descriptive statistics of clinical and demographic characteristics across the three assessment periods Descriptive statistics are presented as mean±SD, median (IQR), or n (%). No statistical tests were applied to this table, as it aims to characterize the study population across the three assessments. n: number of observations; SD: standard deviation; IQR: interquartile range; BP: blood pressure; CVR: cardiovascular risk; HbA1c: glycated hemoglobin; LDL-C: low-density lipoprotein cholesterol; T2DM: type 2 diabetes mellitus

	First assessment	Second assessment	Third assessment
n	270	276	277
Male (n; %)	137 (51.1%)	146 (52.9%)	138 (49.8%)
Age (mean; SD)	68.6±11.3	70.7±10.8	70.5±10.6
Obese (n; %)	76 (28.2%)	73 (26.5%)	81 (29.2%)
Overweight (n; %)	124 (45.9%)	124 (44.9%)	118 (42.6%)
Smokers (n; %)	19 (7%)	26 (9.4%)	43 (15.5%)
Harmful alcohol consumption (n; %)	60 (22.2%)	66 (23.9%)	65 (23.5%)
Systolic BP (mean; SD)	139.9 (±8.0)	138.3 (±15.1)	135.6 (±14.8)
Diastolic BP (mean; SD)	75.6 (±11.3)	75.8 (±10.6)	75.3 (±9.6)
HbA1c (median; IQR)	6.9 (6.3-7.5)	7.2 (6.5-7.9)	7.1 (6.5-7.9)
Duration of T2DM (median; IQR)	9 (4-14)	11 (6-15)	10 (3-15)
Retinopathy (n; %)	29 (10.7%)	32 (11.6%)	28 (10.1%)
Nephropathy (n; %)	20 (7.4%)	53 (19.2%)	54 (19.5%)
Neuropathy (n; %)	3 (1.1%)	3 (1.5%)	8 (2.9%)
Coronary heart disease (n; %)	25 (9.3%)	31 (11.2%)	35 (12.6%)
Cerebral vascular disease (n; %)	14 (5.2%)	16 (5.8%)	21 (7.6%)
Peripheral arterial disease (n; %)	9 (3.3%)	24 (8.7%)	28 (10.1%)
LDL-C (median; IQR)	83 (65.8-105)	77 (63.6-99)	75.3 (57.9-100.2)
High CVR (n; %)	188 (69.6%)	155 (56.2%)	158 (57%)
Very high CVR (n; %)	82 (30.4%)	121 (43.8%)	119 (43%)

Lipid control

In the first assessment, only 55 (20.4%) patients exhibited lipid control within targets, while the majority, 215 (79.6%), did not meet the recommended values. When stratified by CVR, 45 (23.9%) patients with high CVR and 10 (12.2%) with very high CVR achieved the established lipid thresholds.

At the time of the second assessment, following the implementation of CQI interventions, the number of patients achieving lipid goals increased slightly to 60 (21.7%). Among those at high CVR, 35 (22.6%) were within the target, and in the very high CVR group, control improved to 25 (20.7%). Overall lipid control remained suboptimal, with a small improvement of 1.3 percentage points between the first and second assessments, classified as "insufficient" according to the predefined quality standards.

By the third assessment, lipid control improved significantly in the high CVR subgroup, rising to 61 (38.6%), while in the very high CVR group, 29 (24.4%) attained the recommended thresholds. Overall, 90 (32.5%) patients met the defined lipid management goals, an increase of 12.1 percentage points compared to the initial evaluation (from 20.4% to 32.5%). This result corresponds to a "good" quality standard (30-34.9%), reflecting the impact of the implemented interventions. This improvement was confirmed by inferential statistical analysis, which demonstrated a statistically significant increase in lipid control before and after the intervention (95% CI: 4.7-19.3%; p<0.001) (Table 4). 

**Table 4:** Lipid control by CVR group: comparison between the first and third assessments Comparison of lipid control rates between the first and third assessments, categorized by CVR group. Values are presented as n (%). Statistical significance and differences in proportions were assessed using the z-test for independent proportions. The reported values include the z statistic and p-value (p<0.05 considered significant). CVR: cardiovascular risk

CVR group	First assessment (n, %)	Third assessment (n, %)	Difference in proportions (95% CI)	z-test value	P-value
High	45 (23.9%)	61 (38.6%)	4.8-24.2%	-2.949	0.003
Very high	10 (12.2%)	29 (24.4%)	1.2-22.2%	-2.145	0.032
Total	55 (20.4%)	90 (32.5%)	4.7-19.3%	-3.211	<0.001

Additionally, a significant reduction in median LDL-C values was observed (p<0.001), reinforcing the effectiveness of the CQI strategies implemented (Table 5).

**Table 5:** Comparison of median LDL-C levels between the first and third assessments Comparison of median LDL-C levels between the first and third assessments using the Mann-Whitney U test. Values are reported as medians and IQR. The reported values include the U statistic and p-value (p<0.05 considered significant). LDL-C: low-density lipoprotein cholesterol; IQR: interquartile ranges

	LDL-C median (mg/dL)	IQR (mg/dL)	Mann-Whitney U value	P-value
First assessment	83	65.8-105	30811.5	<0.001
Third assessment	75.3	57.9-100.2		

Lipid-lowering treatment and therapeutic inertia

Throughout the three assessments, it was clear that the patients who did not achieve lipid targets consistently were under suboptimal statin therapy (Table 6), particularly high-intensity statins at low doses and moderate-intensity statins. The most frequent therapeutic approach across evaluations involved prescribing high-intensity statins at low doses or moderate-intensity statins at varying doses, reflecting a strategy that does not meet guideline standards. Furthermore, a significant proportion of patients remained without any pharmacological lipid-lowering therapy: 54 (25.2%) in the first assessment, decreasing to approximately 24-29 (11.1-15.5%) in subsequent evaluations. Combined therapy with ezetimibe remained infrequent throughout.

**Table 6:** Top five therapeutic strategies for dyslipidemia across assessments in patients not achieving lipid targets Descriptive data are shown for the top five therapeutic approaches used during each assessment in patients not meeting lipid goals. Values are presented as n (%).

	Therapeutic strategy			(n, %)
First assessment	No pharmacological therapy			54 (25.2%)
	Moderate-intensity statin (low dose)			53 (24.8%)
	Moderate-intensity statin (high dose)			48 (22.4%)
	High-intensity statin (low dose)			30 (14%)
	High-intensity statin (high dose)			7 (3.3%)
Second assessment	High-intensity statin (low dose)			51 (23.6%)
	Moderate-intensity statin (high dose)			37 (17.1%)
	Moderate-intensity statin (low dose)			33 (15.3%)
	No pharmacological therapy			24 (11.1%)
	Combination of high-intensity statin (low dose) and ezetimibe			12 (5.6%)
Third assessment	High-intensity statin (low dose)			38 (20.3%)
	Moderate-intensity statin (high dose)			32 (17.1%)
	No pharmacological therapy			29 (15.5%)
	Moderate-intensity statin (low dose)			22 (11.8%)
	Combination of high-intensity statin (low dose) and ezetimibe			9 (4.8%)

Therapeutic inertia, assessed exclusively among patients with poor lipid control who neither refused treatment nor exhibited poor adherence, was initially very high, affecting 199 (93.9%) patients. However, after the implementation of CQI strategies, this proportion decreased significantly to 154 (76.6%) in the second assessment and 104 (73.8%) in the third, indicating a statistically significant reduction in therapeutic inertia (95% CI: 12.5-28.6%; p<0.001). Furthermore, 37 (17.1%) patients who did not achieve therapeutic targets and for whom no medication adjustment was made had a history of at least one cardiovascular event in the second assessment and 32 (17.1%) in the third. Additionally, 46 (21.3%) of these patients in the second assessment and 40 (21.7%) in the third were over 80 years of age, which may have influenced clinical decisions regarding treatment intensification (Table 7).

**Table 7:** Comparison of therapeutic inertia differences between the first and third assessments Comparison of therapeutic inertia between the first and third assessments using the z-test for independent proportions. The reported values include the z statistic and p-value (p<0.05 considered significant).

	Therapeutic inertia (%)	z-test value	P-value
First assessment	93.9%	5.339	<0.001
Third assessment	73.8%		

Poor therapeutic adherence, initially documented in only two (0.7%) patients, rose to 23 (8.3%) in the second and 27 (9.7%) in the third assessment. Similarly, treatment refusal increased from two (0.7%) to seven (2.9%) across the three assessments, and statin intolerance was reported in only one patient. Among the reasons for non-adherence or refusal, the majority were either unknown or multifactorial. In the qualitative analysis, the most frequently reported causes included lack of awareness regarding the risks associated with the consequences of uncontrolled disease, despite explanations provided by healthcare professionals, fear of side effects, difficulties managing complex treatment regimens, and lifestyle-related decisions. Cost-related barriers and forgetfulness in taking medication were rarely mentioned, accounting for a minimal proportion of cases.

## Discussion

This CQI study demonstrates a statistically significant improvement in lipid control among individuals with T2DM following a structured, multifaceted intervention. The proportion of patients achieving LDL-C or non-HDL-C targets, according to their CVR classification, increased from 20.4% at baseline to 32.5% in the final assessment, meeting the predefined criteria for a "good" quality standard. These findings align with international and national studies reporting persistent gaps in lipid target achievement among patients with diabetes. A Turkish cross-sectional study demonstrated that only 24.8% of statin-treated diabetic patients achieved recommended LDL-C targets [11], while another German-Austrian study revealed even lower attainment rates of 16.3% and 11.8% among high- and very-high-risk individuals, respectively [12]. Similarly, a nationwide study in Korea found that only 26.6% of patients with established CVD and 15.7% of those with multiple risk factors achieved LDL-C targets, with most patients receiving only statin monotherapy [15]. In Portugal, comparable CQI interventions across six FHUs showed an increase in lipid control rates to 32.1% [13]. However, these outcomes were assessed based on previous clinical guidelines, which used more lenient lipid targets. Differences in lipid control outcomes across studies may reflect contextual variations, such as differences in baseline CVR profiles, health system structure, or access to combination therapies.

The comparability of outcomes reinforces the potential for generalizing and scaling such interventions within the Portuguese National Health Service. The success of this intervention model, performance feedback, clinical reminders to support both clinician decision-making and patient engagement, team training, and collaborative goal-setting, is consistent with international multifactorial approaches for diabetes and CVR management [4,9,16]. The J-DOIT3 randomized controlled trial demonstrated that intensified multifactorial interventions targeting lipid control, blood pressure, and glycemia significantly reduced cerebrovascular events [16]. These findings, along with our own, emphasize the cumulative benefit of modest yet consistent improvements in lipid parameters. Current guidelines advocate a "lower is better" LDL-C strategy, especially for patients with at least high CVR [4,9]. 

Despite the progress achieved, optimal lipid control in primary care remains a significant challenge, with nearly two-thirds of the population not reaching recommended targets. Although therapeutic inertia decreased significantly, falling from 93.9% to 73.8%, it remains a common barrier. This finding is consistent with the Swedish national study, which reported that approximately 40% of eligible patients were not prescribed any lipid-lowering therapy [17]. The limited use of combination therapy in the current study, particularly concerning ezetimibe, mirrors the pattern reported in the ALERT-LDL study in Italy, where an intensive, guideline-based approach increased LDL-C target attainment from 9% to 68.5% within 12 months [18]. Further insights from Paul et al. indicate that therapeutic inertia in lipid management correlates with poor long-term cardiovascular outcomes [19]. 

The persistence of suboptimal treatment intensification was reaffirmed by another Portuguese study, which demonstrated ongoing lipid target non-achievement despite structured primary care management [20]. Although the lack of therapeutic intensification may occasionally be justified in older adults (>80 years), it is particularly concerning in patients with prior cardiovascular events who remain uncontrolled. Indeed, the data identified such patients, underscoring a critical gap in care requiring urgent attention to prevent recurrent cardiovascular events. Literature-identified barriers, including prescriber uncertainty, systemic constraints, and patient adherence issues, highlight the need for targeted strategies to address specific clinical, educational, and systemic barriers to therapy optimization [21].

Some clinical variables showed notable changes across assessment periods, as previously shown in Table 3. The prevalence of microvascular complications, such as nephropathy, increased from 7.4% to 19.2%. Similarly, macrovascular complications became more frequently documented: coronary heart disease rose from 9.3% to 12.6% and peripheral arterial disease from 3.3% to 10.1%. In parallel, the proportion of patients classified as having very high CVR increased from 30.4% to 43%. These shifts may reflect improved diagnostic coding, greater clinical awareness, or true worsening of patient profiles over time. A substantial increase in documented smoking prevalence (from 7% to 15.5%) was also observed, possibly due to more systematic assessment and recording during consultations.

Furthermore, the progressive increase in documented poor adherence and treatment refusal may not necessarily reflect a true worsening of patient compliance. Instead, it could possibly be attributed to improved awareness, recognition, and documentation of these behaviors by healthcare providers. This potential shift in reporting practices is clinically relevant, as non-adherence remains a major barrier to effective lipid management. Qualitative data suggested that patient-related factors such as fear of side effects, low health literacy, and lifestyle constraints contributed significantly to non-adherence. Importantly, achieving very low LDL-C levels is not associated with increased harm, but rather with continued CVR reduction [22]. This supports not only the safety of intensifying lipid-lowering therapy when clinically indicated but also the importance of addressing both clinician hesitation and patient fears. Reassurance is crucial to overcoming resistance to more aggressive lipid management and achieving long-term cardiovascular benefit. Additionally, more consultation time is needed to understand and demystify patients' beliefs about medication. Family medicine is a complex specialty in which it is often not possible to address all of a patient's issues within a single appointment.

Lastly, the observed reduction in median LDL-C levels, accompanied by a rise in therapeutic adjustments, indicates a shift towards more proactive management. The structured, team-based CQI approach demonstrated here aligns with international best practices and underscores its potential to sustainably enhance chronic disease outcomes in primary care settings [23,24]. Although CQI interventions likely contributed to the observed improvements, external factors such as broader policy changes, public health campaigns, or increased availability of generics over the study period could have also played a role. These potential confounders were not controlled for and should be explored in future research.

This study has several limitations that should be acknowledged. First, data collection was conducted by multiple investigators, which may have introduced inter-observer variability and information bias, despite the use of a standardized protocol. Second, the retrospective nature of the data and reliance on clinical records may have led to the misclassification of therapeutic inertia; in some cases, the absence of medication changes could reflect justified clinical decisions not documented. Although professionals were encouraged to record reasons for non-intensification, any unreported information cannot be excluded. Also, the exploratory analysis of non-adherence and treatment refusal relied on clinical documentation and was not based on standardized questionnaires or validated adherence tools. As such, the findings may be subject to underreporting, inconsistent recording, and potential recall or interpretation bias. Third, the possibility of a Hawthorne effect, whereby awareness of being observed may have influenced provider behavior, cannot be excluded. The implementation of feedback and visible performance indicators may have temporarily improved clinician engagement, potentially overestimating the long-term impact of the intervention. Furthermore, updates to international guidelines during the study period may have influenced clinical decisions and target classifications. The SCORE2-Diabetes risk model from the 2023 ESC guidelines [25], which estimates 10-year CVR in T2DM patients, was not applied in this study. Additionally, the recommended ≥50% reduction from baseline LDL-C levels was not included as a treatment target due to the frequent unavailability of untreated baseline values, which may have led to the overestimation of therapeutic success in some patients. Finally, as the study was conducted in a single FHU, the results may not fully represent the general Portuguese diabetic population. However, the unit's organizational structure, electronic health record system, and clinical protocols are consistent with national standards, which supports broader applicability. Still, regional differences in resources or staffing may affect scalability, and adaptation could be required in settings with different baseline capacities.

These limitations may have led to the under- or overestimation of performance indicators but do not undermine the overall trends or the clinical relevance of the intervention, which remains one of the few published studies in Portugal evaluating lipid management outcomes in patients with T2DM based on the most recent clinical guidelines advocating stricter lipid targets. The study was conducted over a two-year period, allowing for the evaluation of medium-term trends. Moreover, the data collection process was highly comprehensive, encompassing a broad range of clinical, biochemical, and therapeutic variables, thereby increasing the validity of the findings. However, further follow-up is needed to assess whether the observed improvements are sustained over time and whether they ultimately translate into better cardiovascular outcomes.

## Conclusions

This CQI study highlights that team-based, structured interventions can effectively improve lipid control in patients with T2DM, particularly those at high or very high CVR. The increase in the proportion of patients achieving LDL-C or non-HDL-C targets, alongside the reduction in therapeutic inertia and improvement in clinical documentation relating to poor adherence, reflects meaningful progress in the management of dyslipidemia in primary care. These findings underline ongoing challenges, suboptimal treatment intensification, low adherence, and patient refusal, while reinforcing the importance of implementing strategies that address both clinical decision-making and patient engagement. The safety of intensive lipid-lowering therapy, supported by current evidence, should reassure both clinicians and patients and promote a more proactive therapeutic approach.

To maximize the impact of this intervention, continued implementation and follow-up over the coming years are recommended, in alignment with evolving clinical guidelines. Monitoring should focus on sustained LDL-C control rates, persistence of therapeutic adjustments, and patient adherence. To further validate and expand these findings, future studies should consider multicenter approaches, which could enhance representativeness across different geographical regions and healthcare settings. However, such expansion will require adaptation to local resource availability, baseline CVR profiles, and system-level variability. Expanding the scope to include specific patient subgroups, such as non-diabetic patients at high or very high CVR, may further contribute to improved lipid management and CVR reduction. In parallel, exploring the underlying causes of therapeutic inertia may lead to more reflective and effective prescribing practices. 

This initiative also has the potential to drive CQI in primary care by promoting accurate clinical registers and documentation, appropriate CVR stratification, interdisciplinary collaboration, and optimization of lipid-lowering therapy. Future strategies should incorporate patient-centered interventions, such as targeted education and motivational support, to address behavioral barriers to adherence. The use of technology, including clinical decision support systems and prompts integrated into electronic health records, may further support therapeutic decision-making and help reduce inertia. Although the results are promising, they derive from a single FHU, which may limit generalizability. Lastly, future research should assess the long-term impact of these interventions not only on lipid parameters but also on hard cardiovascular outcomes, to fully demonstrate their clinical benefit.

## References

[1] Fundação Francisco Manuel dos Santos (2025). PORDATA: deaths by selected causes (%). https://www.pordata.pt/sites/default/files/2024-06/Portugal_Obitos_por_algumas_causas_de_morte_%28%25%29.xlsx.

[2] (2025). National Program for Cerebrocardiovascular Diseases. Lisbon: DGS.

[3] (2025). Integrated care pathway for cardiovascular risk in adults. https://www.dgs.pt/documentos-e-publicacoes/processo-assistencial-integrado-do-risco-cardiovascular-no-adulto-pdf.aspx.

[4] Visseren FL, Mach F, Smulders YM (2021). 2021 ESC guidelines on cardiovascular disease prevention in clinical practice. Eur Heart J.

[5] Ferreira L, Rodrigues M (2015). Study double D: diabetes and dyslipidemia. Rev Port Diabetes.

[6] Bahiru E, Hsiao R, Phillipson D, Watson KE (2021). Mechanisms and treatment of dyslipidemia in diabetes. Curr Cardiol Rep.

[7] Raghavan S, Vassy JL, Ho YL (2019). Diabetes mellitus-related all-cause and cardiovascular mortality in a national cohort of adults. J Am Heart Assoc.

[8] Parizad R, Batta A, Hatwal J, Taban-Sadeghi M, Mohan B (2025). Emerging risk factors for heart failure in younger populations: a growing public health concern. World J Cardiol.

[9] ElSayed NA, Aleppo G, Aroda VR (2023). Cardiovascular disease and risk management: Standards of Care in Diabetes-2023. Diabetes Care.

[10] Ray KK, Haq I, Bilitou A (2023). Treatment gaps in the implementation of LDL cholesterol control among high- and very high-risk patients in Europe between 2020 and 2021: the multinational observational SANTORINI study. Lancet Reg Health Eur.

[11] Bayram F, Sonmez A, Haymana C (2020). Utilization of statins and LDL-cholesterol target attainment in Turkish patients with type 2 diabetes - a nationwide cross-sectional study (TEMD dyslipidemia study). Lipids Health Dis.

[12] Brandts J, Tittel SR, Bramlage P (2023). Low-density lipoprotein cholesterol and non-high-density lipoprotein cholesterol in type 1 diabetes and type 2 diabetes: lipid goal attainment in a large German-Austrian diabetes registry. Diabetes Obes Metab.

[13] Rafael A, Santos AM, Carneiro C (2021). Diabetes: cardiovascular risk and target LDL in users of six family health units. AIMGF Mag.

[14] Grundy SM, Stone NJ, Bailey AL (2019). 2018 AHA/ACC/AACVPR/AAPA/ABC/ACPM/ADA/AGS/APhA/ASPC/NLA/PCNA guideline on the management of blood cholesterol: executive summary: a report of the American College of Cardiology/American Heart Association Task Force on Clinical Practice Guidelines. J Am Coll Cardiol.

[15] Yun SJ, Jeong IK, Cha JH (2021). Current status of low-density lipoprotein cholesterol target achievement in patients with type 2 diabetes mellitus in Korea compared with recent guidelines. Diabetes Metab J.

[16] Ueki K, Sasako T, Okazaki Y (2017). Effect of an intensified multifactorial intervention on cardiovascular outcomes and mortality in type 2 diabetes (J-DOIT3): an open-label, randomised controlled trial. Lancet Diabetes Endocrinol.

[17] Karlsson SA, Franzén S, Svensson AM, Miftaraj M, Eliasson B, Andersson Sundell K (2018). Prescription of lipid-lowering medications for patients with type 2 diabetes mellitus and risk-associated LDL cholesterol: a nationwide study of guideline adherence from the Swedish National Diabetes Register. BMC Health Serv Res.

[18] Bosso G, De Luca M, Alma G (2022). ALERT-LDL: adherence to guidelines in the treatment of patients with dyslipidemia. Intern Emerg Med.

[19] Paul SK, Klein K, Thorsted BL, Wolden ML, Khunti K (2015). Delay in treatment intensification increases the risks of cardiovascular events in patients with type 2 diabetes. Cardiovasc Diabetol.

[20] Pais C, de Pinho R (2023). Prevalence of people with diabetes who meet therapeutic targets in different risk factors - the reality of a list. Revista Portuguesa de Hipertensão e Risco Cardiovascular.

[21] Zullig LL, Egbuonu-Davis L, Trasy A, Oshotse C, Goldstein KM, Bosworth HB (2018). Countering clinical inertia in lipid management: expert workshop summary. Am Heart J.

[22] Olsson AG, Angelin B, Assmann G (2017). Can LDL cholesterol be too low? Possible risks of extremely low levels. J Intern Med.

[23] Banach M, Surma S, Reiner Z (2022). Personalized management of dyslipidemias in patients with diabetes-it is time for a new approach (2022). Cardiovasc Diabetol.

[24] Soroush N, Nekouei Shahraki M, Mohammadi Jouabadi S, Amiri M, Aribas E, Stricker BH, Ahmadizar F (2024). Statin therapy and cardiovascular protection in type 2 diabetes: the role of baseline LDL-cholesterol levels. A systematic review and meta-analysis of observational studies. Nutr Metab Cardiovasc Dis.

[25] Marx N, Federici M, Schütt K (2023). 2023 ESC guidelines for the management of cardiovascular disease in patients with diabetes. Eur Heart J.

